# Supplementary material for: Progress and challenges in achieving tuberculosis elimination in India by 2025: A systematic review and meta-analysis
Source: PLoS One. 2024 Mar 27;19(3):e0301060. doi: 10.1371/journal.pone.0301060 (PMC10971764; doi:10.1371/journal.pone.0301060)
Supplement: S1 File — (DOCX) [file pone.0301060.s002.docx]

# Search Strategies

## MEDLINE (PubMed) Search Strategy

Database: MEDLINE (PubMed)
Search Date: October 20, 2023

("Tuberculosis"[MeSH Terms] OR "TB" OR "Mycobacterium tuberculosis"[All Fields])
AND
("India"[MeSH Terms] OR "Indian"[All Fields])
AND
("Elimination"[MeSH Terms] OR "Eradication"[All Fields] OR "Control"[All Fields] OR "Prevention"[All Fields])
AND
("Interventions"[MeSH Terms] OR "Treatments"[All Fields] OR "Programs"[All Fields] OR "Strategies"[All Fields] OR "Policies"[All Fields])
AND
("Effectiveness"[MeSH Terms] OR "Efficacy"[All Fields] OR "Outcomes"[All Fields] OR "Impact"[All Fields])

## Embase Search Strategy

Database: Embase (via Ovid)
Search Date: October 20, 2023

('tuberculosis'/exp OR 'tb' OR 'mycobacterium tuberculosis')
AND
('india'/exp OR 'indian')
AND
('elimination' OR 'eradication' OR 'control' OR 'prevention')
AND
('intervention'/exp OR 'treatment' OR 'program' OR 'strategy' OR 'policy')
AND
('effectiveness' OR 'efficacy' OR 'outcome'/exp OR 'impact')

## Web of Science Search Strategy

Database: Web of Science
Search Date: October 20, 2023

TS=(("Tuberculosis" OR "TB" OR "Mycobacterium tuberculosis")
AND
("India" OR "Indian")
AND
("Elimination" OR "Eradication" OR "Control" OR "Prevention")
AND
("Interventions" OR "Treatments" OR "Programs" OR "Strategies" OR "Policies")
AND
("Effectiveness" OR "Efficacy" OR "Outcomes" OR "Impact"))

## Scopus Search Strategy

Database: Scopus
Search Date: October 20, 2023

(TITLE-ABS-KEY ( "Tuberculosis" OR "TB" OR "Mycobacterium tuberculosis" )
AND
TITLE-ABS-KEY ( "India" OR "Indian" )
AND
TITLE-ABS-KEY ( "Elimination" OR "Eradication" OR "Control" OR "Prevention" )
AND
TITLE-ABS-KEY ( "Interventions" OR "Treatments" OR "Programs" OR "Strategies" OR "Policies" )
AND
TITLE-ABS-KEY ( "Effectiveness" OR "Efficacy" OR "Outcomes" OR "Impact" ))
